# A scoring model for preoperative differentiation of high-enhancement pancreatic ductal adenocarcinoma from mass-forming chronic pancreatitis

**DOI:** 10.1186/s12880-025-01830-x

**Published:** 2025-07-18

**Authors:** Jiaping Zhou, Xiaojie Wang, Haifeng Zhang, Yao Pan, Weilin Wang, Risheng Yu

**Affiliations:** 1 Department of Radiology, The Second Affiliated Hospital, School of Medicine, Zhejiang University, Jiefang Road 88#, Hangzhou, 310009 Zhejiang Province China; 2 Department of Radiology, Hangzhou Ninth People’s Hospital, Yilong Road 98#, Hangzhou, 311225 Zhejiang Province China; 3 Department of Hepatobiliary and Pancreatic Surgery, The Second Affiliated Hospital, School of Medicine, Zhejiang University, Jiefang Road 88#, Hangzhou, Zhejiang Province China

**Keywords:** Mass-forming chronic pancreatitis, Pancreatic ductal adenocarcinoma, Magnetic resonance imaging, Scoring model

## Abstract

**Background:**

The present study aimed to establish a scoring model for the differential diagnosis of high-enhancement pancreatic ductal adenocarcinoma (hPDAC) versus mass-forming chronic pancreatitis (MFCP).

**Methods:**

A retrospective analysis was conducted on 81 patients: 40 with MFCP and 41 with hPDAC. Demographic and imaging characteristics were collected. Univariate, ridge regression and binary logistic regression analyses were performed to identify independent predictors and develop diagnostic models. The clinicoradiological model was subsequently converted into a weighted scoring model. Calibration tests, receiver operating characteristic (ROC) curves, area under the ROC curve (AUC), and cut-off points were assessed for both the clinicoradiological and scoring models.

**Results:**

Four independent predictors were included in the clinicoradiological model: lesion size (*p* = 0.012), carbohydrate antigen 19 − 9 (CA19-9) elevate (*p* = 0.003), irregular lesion shape (*p* = 0.024), and pancreatic duct cut-off (*p* = 0.003). Weighted scores were assigned as follows: CA19-9 elevate, 6 points; smaller lesion size, 2 points; irregular lesion shape, 2 points; and pancreatic duct cut-off, 7 points. The clinicoradiological model and the scoring model exhibited AUC values of 0.986 and 0.940, respectively, revealed no significantly difference observed between the two (*p* = 0.073, DeLong test). The scoring model was stratified into two ranges: 0–8 points indicating MFCP and 9–17 points indicating hPDAC.

**Conclusions:**

A concise and practical scoring model for differentiating hPDAC from MFCP was developed, demonstrating strong diagnostic performance. A score of 8 points serves as the key demarcation line in this model.

**Supplementary Information:**

The online version contains supplementary material available at 10.1186/s12880-025-01830-x.

## Background

Pancreatic ductal adenocarcinoma (PDAC) accounts for the majority (approximately 90%) of pancreatic neoplasms [1]. Its incidence is rising at an annual rate of 0.5–1.0%, and by 2023, pancreatic cancer had become the fourth-leading cause of cancer-related mortality [1, 2]. Although the 5-year survival rate has shown a modest increase, it still only approaches 12% [3, 4]. Most patients with PDAC present are diagnosed at an advanced-stage, and only 15–20% are considered candidates for surgical resection [2, 5, 6].

PDAC typically presents with recurrent abdominal pain, weight loss, and imaging evidence of lesions. Because of the poor blood supply, the imaging enhancement of PDAC tends to be weaker than pancreatic parenchyma, often showing peripheral enhancement with central hypo-enhancement [7–9]. Mass-forming chronic pancreatitis (MFCP) has always been one of the most important differential diagnoses of PDAC because of its similar clinical presentation and imaging features, although the two diseases differ significantly in both treatment approaches and prognosis.

In most cases, PDAC and MFCP can be differentiated by their distinct enhancement patterns and degrees of enhancement. However, in both literature reports and clinical practice, we have observed that some cases of PDAC display atypical enhancement during contrast-enhanced scanning, presenting as isometric enhancement to the pancreatic parenchyma in the computed tomography (CT) pancreatic phase, and equal or slightly higher enhancement on magnetic resonance imaging (MRI) [10–13]. This enhancement pattern likely reflects histopathological heterogeneity within the mass, characterized by hypercellular tumor regions interspersed with inflammatory infiltrates. The inflammatory component may arise from secondary peritumoral inflammation induced by pancreatic adenocarcinoma or from malignant transformation of pre-existing chronic pancreatitis [14, 15]. Because of these overlapping radiological features, distinguishing between these conditions remains a persistent diagnostic challenge. Although some studies have noted the existence of PDAC with such atypical enhancement, relevant dedicated research has not been reported. Therefore, in this study, we collected cases of PDAC showing higher enhancement than pancreatic parenchyma in the portal venous phase and provisionally defined this subtype as high-enhancement PDAC (hPDAC). Accurate diagnosis and differential diagnosis are essential to avoid delays caused by misdiagnosis and to prevent unnecessary surgical treatment.

Endoscopic ultrasound-guided fine-needle aspiration biopsy (EUS-FNA) can accurately obtain cytopathological evidence from pancreatic masses, with reported sensitivity, specificity, and accuracy ranging from 79 to 98%, 71–100%, and 82–98%, respectively [16–19]. Despite its effectiveness, the existence of a certain degree of false-negative rate may also delay the diagnosis and management. A previous study demonstrated that the presence of chronic pancreatitis significantly reduced the detection rate of pancreatic malignant tumors by EUS-FNA, showing sensitivity, specificity, and accuracy of 54%, 100%, and 91%, respectively [20, 21]. Consequently, noninvasive imaging examinations plays an important role in differentiating pancreatic cancer from chronic pancreatitis. CT and MRI remain the cornerstone for distinguishing PDAC from MFCP, with MRI offering the dual advantages of avoiding ionizing radiation and providing superior soft tissue contrast resolution. MRI outperforms CT in visualizing normal pancreatic tissue and granulation tissue within lesions. The NCCN 2020 Guidelines for PDAC indicate that MRI can detect PDAC lesions not visible on CT scans [22, 23].

Therefore, we established differential diagnosis models for hPDAC and MFCP based on clinical information and MR imaging features to analyze and compare their diagnostic efficacy. Furthermore, we developed a weighted scoring system for the clinicoradiological model to ensure both discriminatory capacity and concise evaluation.

## Method

### Patients

**Fig. 1:**
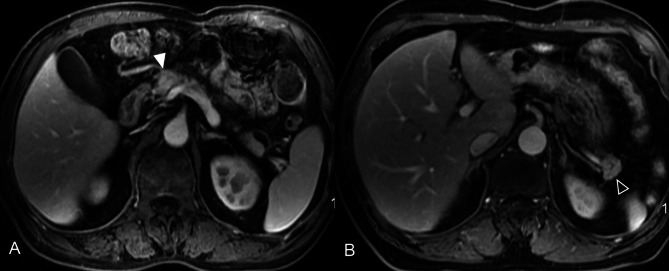
MRI of high-enhancement pancreatic ductal adenocarcinoma and mass-forming chronic pancreatitis. (**A**) Pancreatic ductal adenocarcinoma showed the higher enhancement than pancreatic parenchyma in portal venous phase (white narrow); (**B**) Mass-forming chronic pancreatitis showed higher enhancement than pancreatic parenchyma in portal venous phase (hollow narrow)

**Fig. 2:**
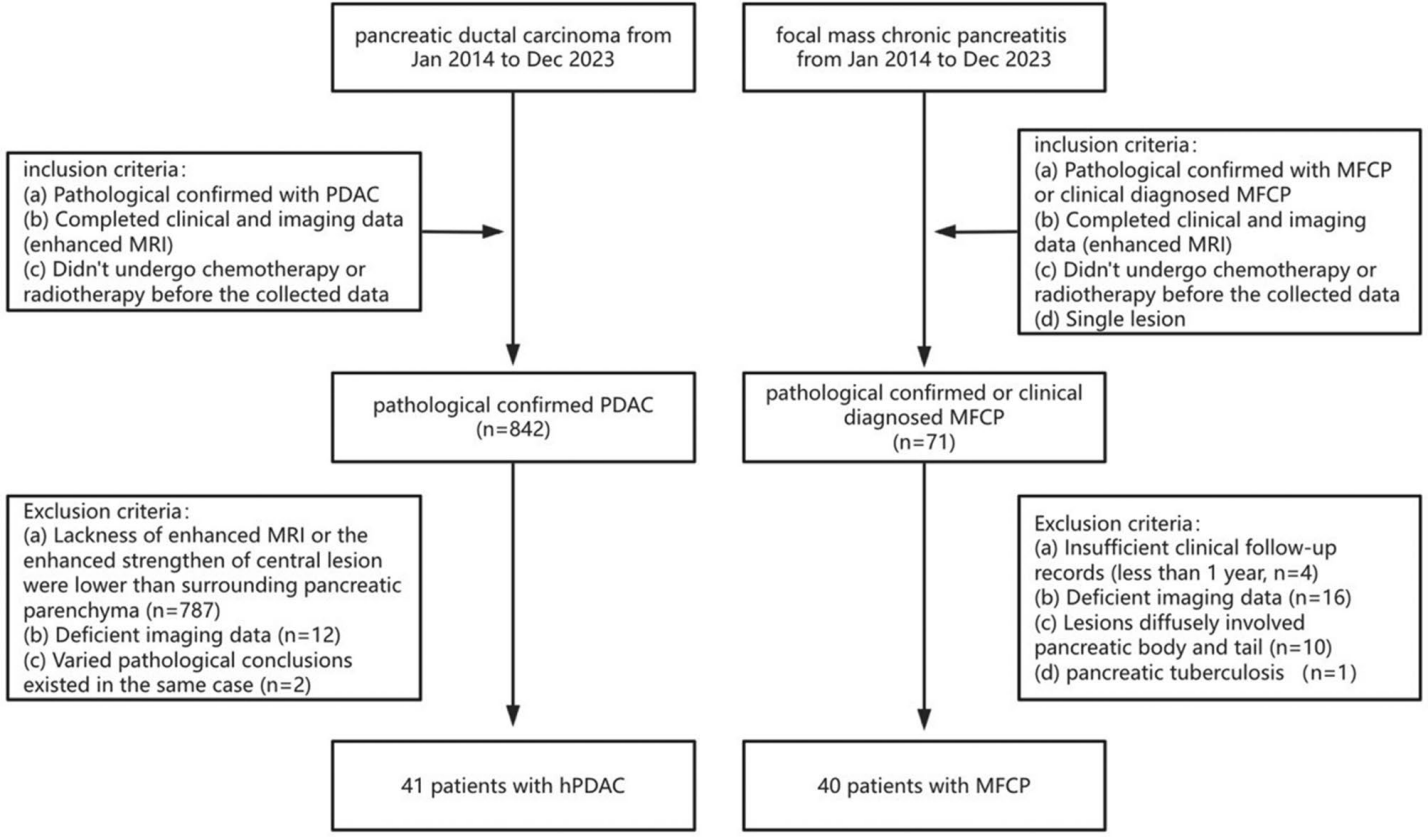
Recruitment pathway for eligible patients in this study

Overall, 81 patients—40 with MFCP and 41 with hPDAC—were enrolled in this retrospective study from the Second Affiliated Hospital of Zhejiang University School of Medicine. All patients were diagnosed between January 2014 and December 2023. The enhancement pattern of hPDAC observed in this study is shown in Fig. 1. The inclusion criteria hPDAC and MFCP were a confirmed pathological diagnosis or clinical diagnosis MFCP clinical data and preoperative enhanced MRI, no prior chemotherapy or radiotherapy before data collection, and the presence of a single lesion in MFCP cases. For hPDAC, the exclusion criteria were absence of enhanced MRI or lesions with enhancement lower than the surrounding pancreatic parenchyma (*n* = 787), deficient imaging data (*n* = 12), and inconsistent pathological conclusions (*n* = 2). For MFCP, the exclusion criteria were insufficient clinical follow-up records of less than 1 year (*n* = 4), deficient imaging data (*n* = 16), diffuse lesion involved pancreatic body and tail (*n* = 10), and diagnosis of pancreatic tuberculosis (*n* = 1), as detailed in Fig. 2.

### MR imaging acquisition

MRI examinations were conducted with a 3-Tesla scanner (Discovery MR750; GE Healthcare). All patients underwent axial imaging without respiratory motion artifacts, acquiring fat-saturated T1-weighted, T2-weighted, and multi-phase contrast-enhanced T1-weighted sequences. Gadobutrol (Omniscan; GE Healthcare, 0.1 mL/kg body weight) was intravenously administered at a rate of 2 mL/s, and dynamic contrast-enhanced T1-weighted imaging began 20 s after contrast injection, with multiphasic acquisitions including arterial (20 s), portal venous (45–52 s), equilibrium (75–82 s), and delayed (135–142 s) phases, using a slice thickness/gap of 4/2 mm.

### Image analysis

Imaging features were evaluated in the portal phase by two radiologists, both of whom were blinded to the pathological results. Any disagreements were resolved by consensus with a third radiologist with 32 years’ experience in abdominal imaging.

Demographic variables including sex, age, serum carbohydrate antigen 19 − 9 (CA19-9), and carcinoembryonic antigen (CEA) were recorded. Radiological features were assessed based on lesion location (pancreatic head, neck, body, or tail), size (maximal axial diameter), shape (defined as regular if > 80% of the lesion’s contour demonstrated a smooth oval or circular outline, otherwise irregular), lobulation (presence of multiple curved processes alternating with depressions), and margin (considered well-defined if > 80% of the lesion showed smooth circumscription without spiculation or infiltration, otherwise ill-defined) [24]. Additional imaging characteristics included cystic degeneration (solid enhancing components comprising < 50% of the lesion), lymphadenopathy (short-axis diameter of > 10 mm or presence of necrosis) [25, 26], liver metastasis (multiple hypo-vascular or peripherally enhancing nodules) [27], anterior renal fascia thickening, peripancreatic vascular invasion (vessel occlusion or venous thrombosis), pancreatic atrophy (parenchymal width less than one-third of vertebral body diameter distal to the lesion) [14, 28], pancreatic duct dilatation (main pancreatic duct diameter of ≥ 3 mm) [29], morphology of the dilated pancreatic duct, pancreatic duct cut-off (abrupt interruption without a complete duct traversing the mass), common bile duct dilatation (diameter of > 8 mm), morphology of the dilated common bile duct, and the presence of a double-duct sign.

### Statistical analysis

The data distribution was assessed using the Kolmogorov–Smirnov or Shapiro–Wilk test. Normally distributed continuous data are expressed as mean ± standard deviation, while non-normally distributed data are presented as median (interquartile range). Categorical data are summarized as frequency and percentage. In univariate analyses, age and lesion size were conducted using the chi-square test or Fisher’s exact test, Student’s t-test or the Mann–Whitney U test for while sex, serum tumor markers and MR imaging features. Significant univariate predictors were subjected to ridge regression to address potential multicollinearity, followed by binary multivariate logistic regression to identify independent predictors. Regression coefficients were transformed into integer scores using a weighting algorithm, which involved dividing the regression coefficient of each predictor by half of the minimum coefficient and then rounding to the nearest integer part or truncating to the integer part to establish optimal scores for the respective features [30, 31]. The discriminatory capacity of models was validated by receiver operating characteristic (ROC) curve analysis. The area under the ROC curve (AUC), optimal cut-off values, along with corresponding specificity and sensitivity, were provided. The DeLong nonparametric method was conducted for AUC comparisons.

Statistical analyses were performed using SPSS version 25.0 (IBM Corp., Armonk, NY, USA), MedCalc version 19.0.4 (MedCalc Software Bvba, Ostend, Belgium) and GraphPad Prism 7.0, a p-value < 0.05 indicated a statistical significance.

## Results

### Comparison of clinical data and imaging features

The baseline data and imaging features of the participants are summarized and present in Tables 1 and 2. MFCP showed a male predominance, while hPDAC occurred slightly more often in men (87.50% [35/40] vs. 60.98% [25/41], *p* = 0.006). Patients with MFCP were significantly younger than those with hPDAC (57.23 ± 13.82 vs. 64.00 ± 7.21 years, *p* = 0.008). Additionally, the incidence of CA19-9 and CEA elevation was significantly lower in MFCP group than in hPDAC group (CA19-9 elevation: 22.50% [9/40] vs. 70.73% [29/41], *p* < 0.001; CEA elevation: 12.50% [5/40] vs. 31.71% [13/41], *p* = 0.038).

**Table 1:** Clinical information between MFCP and hPDAC

Characteristics	*n*	Category		*p*
		MFCP (*n* = 40)	hPDAC (*n* = 41)	
Age (years)	60.65 ± 11.47	57.23 ± 13.82	64.00 ± 7.21	**0.008**
Sex				**0.006**
Male	60(74.10)	35(87.50)	25(60.98)	
Female	21(25.90)	5(12.50)	16(39.02)	
CA19-9 elevate (>37 U/mL)	81			**<0.001**
Yes	38(46.91)	9(22.50)	29(70.73)	
No	43(53.07)	31(77.50)	12(29.27)	
CEA elevate (>5ng/mL)	81			**0.038**
Yes	18(19.18)	5(12.50)	13(31.71)	
No	63(80.82)	35(87.50)	28(68.29)	

**Table 2:** MR imaging features between MFCP and hPDAC

Characteristics	*n*	Category		*p*
		MFCP (*n* = 40)	hPDAC (*n* = 41)	
Lesion size (cm*)	2.75(1.56)	3.53(1.90)	2.35(0.90)	**<0.001**
Location				**0.004**
Head	50(61.73)	18(45.0)	32(78.05)	
Neck	6(7.41)	5(12.50)	1(2.44)	
Body	11(13.58)	5(12.50)	6(14.63)	
Tail	9(11.11)	7(17.50)	2(4.88)	
Multiple	5(6.17)	5(12.50)	0(0)	
Lesion shape				**0.017**
Irregular	51(62.96)	20(50.00)	31(75.61)	
Regular	30(37.04)	20(50.00)	10(24.39)	
Lobulation				**<0.001**
Present	28(34.57)	6(15.00)	22(53.66)	
Absent	53(65.43)	34(85.00)	19(46.34)	
Margin				0.517
well-defined	56(69.14)	29(72.50)	27(65.85)	
Ill-defined	25(30.86)	11(27.50)	14(34.15)	
Cystic degeneration				0.712
Present	8(9.88)	3(7.50)	5(12.20)	
Absent	73(90.12)	37(92.50)	36(87.80)	
Hepatic metastasis				0.494
Present	2(2.47)	0(0)	2(4.88)	
Absent	79(97.53)	40(100.00)	39(95.12)	
Lymphadenopathy				**0.018**
Present	35(43.21)	12(30.00)	23(56.10)	
Absent	46(56.79)	28(70.00)	18(43.90)	
Thickened anterior renal fascia				0.096
Present	11(13.58)	8(20.00)	3(7.32)	
Absent	70(86.42)	32(80.00)	38(92.68)	
Peripancreatic vascular invasion				0.753
Present	9(11.11)	4(10.00)	5(12.20)	
Absent	72(88.89)	36(90.00)	36(88.90)	
Pancreatic atrophy				**<0.001**
Present	48(59.26)	16(40.00)	32(78.05)	
Absent	33(40.73)	24(60.00)	9(21.95)	
Dilated pancreatic duct				**0.010**
Present	48(59.26)	18(45.00)	30(73.17)	
Absent	33(40.74)	22(55.00)	11(28.83)	
Morphology of the dilated pancreatic duct				**0.034**
Normal	46(56.79)	28(70.00)	18(43.90)	
Warp	24(29.63)	9(22.50)	15(36.59)	
Irregularly dilated	11(13.58)	3(7.50)	8(19.51)	
Pancreatic duct cut-off				**<0.001**
Present	31(38.27)	3(7.50)	28(68.29)	
Absent	50(61.73)	37(92.50)	13(31.71)	
Dilated common bile duct				**0.010**
Present	38(46.91)	13(32.50)	25(60.98)	
Absent	43(53.09)	27(67.50)	16(39.02)	
Morphology of the dilated common bile duct				**0.026**
Normal	46(56.79)	27(67.50)	19(46.34)	
Warp	7(8.64)	5(12.50)	2(4.88)	
Irregularly dilated	28(34.57)	8(20.00)	20(48.78)	
Double duct sign				**0.049**
Present	31(38.27)	11(27.50)	20(48.78)	
Absent	50(61.73)	29(72.50)	21(51.22)	

*cm: centimeter

Regarding imaging features, MFCPs were more commonly located in the pancreatic head (45.00%, 18/40), whereas hPDACs were predominantly found in the head as well, but at a higher frequency (78.05%, 32/41). Lesion size was significantly smaller in hPDACs than in MFCPs (23.5 vs. 35.3 mm, *p* < 0.001). Irregular lesion shape and lobulation were both significantly more prevalent in hPDACs than in MFCPs (irregular shape: 75.61% [31/41] vs. 50.00% [20/40], *p* = 0.017; lobulation: 53.66% [22/41] vs. 15.00% [6/40], *p* < 0.001). The most characteristic imaging manifestations of hPDACs were pancreatic atrophy and pancreatic duct cut-off, which occurred at significantly higher rates than in MFCPs (pancreatic atrophy: 78.05% [32/41] vs. 40.00% [16/40]; pancreatic duct cut-off: 68.29% [28/41] vs. 7.50% [3/40]; both *p* < 0.001). Additionally, hPDACs exhibited significantly higher rates of pancreatic duct dilation, common bile duct dilation, and lymphadenopathy than MFCPs (pancreatic duct dilation: 73.17% [30/41] vs. 45.00% [18/40], *p* = 0.010; common bile duct dilation: 60.98% [25/41] vs. 32.50% [13/40], *p* = 0.010; lymphadenopathy: 56.10% [23/41] vs. 30.00% [12/40], *p* = 0.018). By contrast, MFCPs were significantly more likely to show normal morphology of both the pancreatic duct and common bile duct (normal pancreatic duct morphology: 70.00% [28/40] vs. 43.90% [18/41], *p* = 0.034; normal common bile duct morphology: 67.50% [27/40] vs. 46.34% [19/41], *p* = 0.026). No significant differences were found in lesion margin, cystic degeneration, hepatic metastasis, thickening of the anterior renal fascia, or peripancreatic vascular invasion between hPDACs and MFCPs.

### Establishing diagnostic models for differentiation

**Fig. 3:**
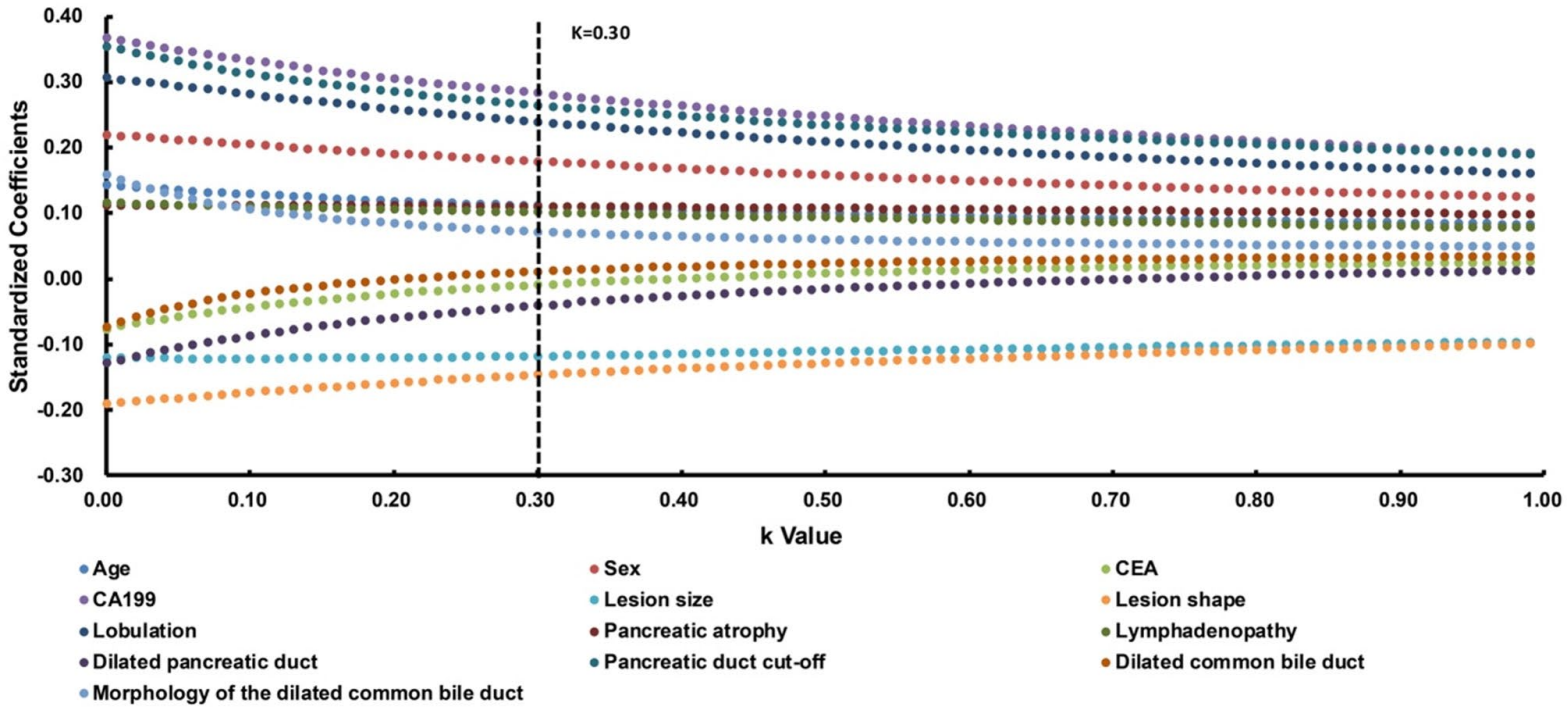
The ridge curve of relevant predictive factors

Based on the prior univariate analyses (*p* < 0.05), significant features were selected by regression analysis for minimizing multicollinearity in the subsequent multivariate model. As illustrated by the ridge trace curve (Fig. 3), a K value of 0.30 produced stable standardized coefficients, confirming the significance of the model (*p* < 0.001). At this threshold, nine factors were positively correlated with the differential diagnosis: age (*p* = 0.028), sex (*p* < 0.001), elevated CA19-9 levels (*p* < 0.001), lesion size (*p* = 0.021), lesion shape (*p* = 0.004), lobulation (*p* < 0.001), pancreatic atrophy (*p* = 0.030), lymphadenopathy (*p* = 0.044), and pancreatic duct cut-off (*p* < 0.001) (Table S1).

Independent predictors for distinguishing h PDAC from MFCP were further validated and determined by multivariate logistic regression analysis (Table S2). In the clinical model, age (*p* = 0.008), male sex (*p* = 0.006), and elevated CA19-9 levels (*p* < 0.001) emerged as significant independent predictors, whereas elevated CEA levels (*p* = 0.405) did not. In the imaging model, lesion size (*p* = 0.042), irregular lesion shape (*p* = 0.015), lobulation (*p* = 0.018), pancreatic atrophy (*p* = 0.046), and pancreatic duct cut-off (*p* = 0.004) were identified as significant independent predictors. Finally, the combined clinicoradiological model confirmed lesion size (*p* = 0.012), elevated CA19-9 levels (*p* = 0.003), irregular lesion shape (*p* = 0.024), and pancreatic duct cut-off (*p* = 0.003) as independent predictors. Details results are presented in Table 3.

**Table 3:** Multivariate logistic regression of clinicoradiological model

	B	*p*	OR	95% CI OR	Weighted score
CA19-9 elevate	7.531	**0.003**	1865.302	13.986–248765.407	6
Lesion Size (smaller)	-2.736	**0.012**	0.065	0.008–0.553	2
Lesion shape (Irregular)	-3.16	**0.024**	0.042	0.003–0.664	2
Pancreatic duct cut-off	9.407	**0.003**	12169.782	25.286–5857195.177	7
Constant	2.808	0.176	16.57	0.284–966.482	

### Establishment of scoring model

A scoring model was derived from the combined clinicoradiological model for quantizing the risk classification. Scores were assigned by dividing the regression coefficient of each predictor by half of the minimum coefficient and then rounding to the nearest integer part or truncating to the integer part. In this study, the smallest coefficient belonged to lesion size, with an absolute value of 2.736; therefore, half of this value (1.368) served as the denominator. The weighted scores for the independent predictors were as follows: elevated CA19-9 levels, 6 points; smaller lesion size, 2 points; irregular lesion shape, 2 points; and pancreatic duct cut-off, 7 points (Table 3). This scoring model produced a maximum total of 17 points, calculated by summing the individual scores associated with all predictors for each patient.

### Performance of clinical model, imaging model, clinicoradiological model, and scoring model

**Fig. 4:**
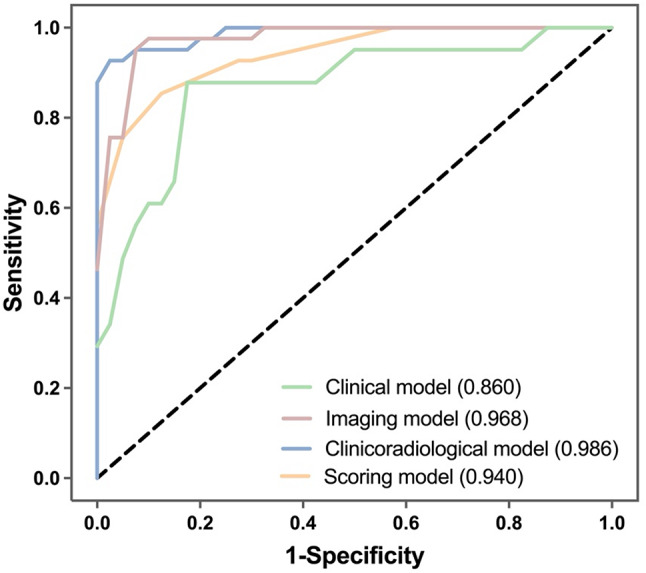
The ROCs of clinical model, imaging model, clinicoradiological model and scoring model

The Hosmer-Lemeshow goodness-of-fit test was used to evaluate the calibration of the logistic model, and the results showed that all models had good calibration (*p* > 0.05) (Figure S1). Based on this satisfactory calibration, predictive performance was assessed using ROC curves (Table 4; Fig. 4). The clinicoradiological model demonstrated high discriminative ability, achieving a sensitivity of 92.7% and specificity of 97.5%. The AUC for this model was 0.986, which was comparable to that of the scoring model (AUC = 0.940), with no significant statistical difference confirmed by the DeLong test (*p* = 0.073). These results suggest that the scoring model effectively retains the discriminative power of the predictive clinicoradiological model while offering a concise and practical tool for the differential diagnosis of hPDAC and MFCP.

**Table 4:** The differential diagnostic efficiency of models

	AUC	*p*	95% CI for OR		Cut-off point (sensitivity, specificity)
			Lower	Upper	
Clinical model	0.858	<0.001	0.774	0.942	0.413 (0.878, 0.825)
Imaging model	0.955	<0.001	0.916	0.993	0.264 (0.951, 0.825)
Clinicoradiological model	0.986	<0.001	0.968	0.998	0.642 (0.927, 0.975)
Scoring model	0.940	<0.001	0.893	0.987	0.729 (0.854, 0.875)

### Score ranges exploration

**Fig. 5:**
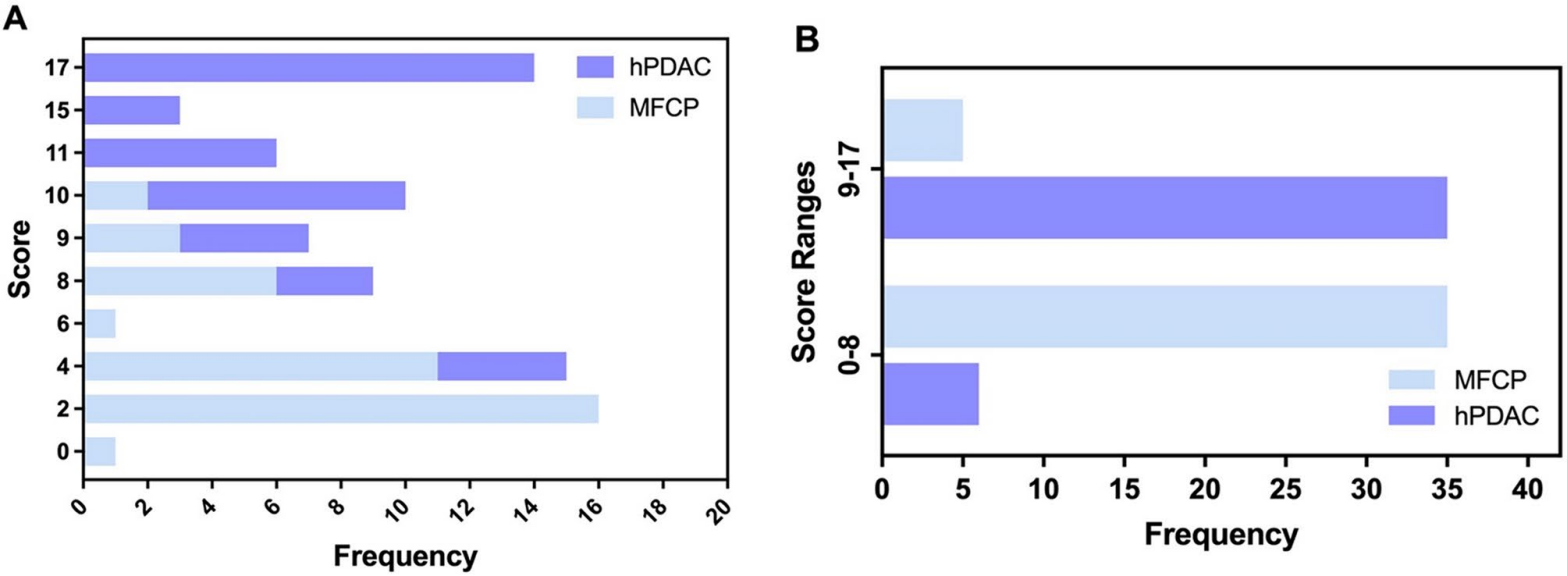
The frequency distribution according to the score assigned of hPDAC and MFCP groups

The scoring model, incorporating four weighted-score features, was systematically applied to all study cases. Based on the frequency distribution, a significant diagnostic cut-off of 8 points was established for distinguishing between the two diseases (Fig. 5a). To facilitate practical application, the scores were divided into two ranges: 0–8 points indicating MFCP and 9–17 points indicating hPDAC. This dichotomization enhances operational efficiency in routine diagnostic workflows while maintaining discriminative accuracy. The frequency distribution across the scoring gradient is illustrated in Fig. 5b, with the corresponding positive predictive rates for these ranges summarized in Table 5.

**Table 5:** The predicted positive rates of scoring model

Score range	Predicted true positive	Actual positive	Precision	Predicted total positive	Recall	F1 score
≥ 0 and ≤ 8	35^1^	40^1^	87.50%	41^1, 2^	85.37%	0.864
>8 and ≤ 17	35^2^	41^2^	85.37%	40^1, 2^	87.50%	0.864

1: MFCP; 2: hPDAC

## Discussion

The successful establishment of a clinicoradiological diagnostic model carries conviction for differentiating hPDAC from MFCP, incorporating four variables—elevated CA19-9 levels, lesion size, lesion shape, and pancreatic duct cut-off—that served as independent predictors. This model demonstrated high diagnostic accuracy, achieving a sensitivity of 92.7%, specificity of 97.5%, and AUC of 0.986. Building on this model, we developed a scoring system using weighted multivariate predictors and stratified the scores into two ranges to distinguish hPDAC from MFCP. Both the diagnostic model and the scoring model demonstrated satisfactory discriminatory performance for the two diseases.

Statistically significant differences were observed in sex; age; CA19-9 and CEA levels; size, location, shape, and lobulation; lymphadenopathy; pancreatic atrophy; and duct characteristics, findings consistent with previous studies [23, 32, 33]. However, the predictive features identified through univariate analyses were not meant to be independent predictors, highlighting the need for further exploration through multiple regression analyses.

After performing ridge regression to alleviate multicollinearity, subsequent multivariate logistic regression analyses confirmed increased CA19-9 levels, lesion size, lesion shape, and pancreatic duct cut-off as robust predictive factors.

Tumor markers play a critical role in differentiating PDAC from MFCP, with CA19-9 is a widely validated biomarker for PDAC. It serves as a predictor of disease stage, prognosis, resectability, recurrence, and therapeutic response [34–36]. CA19-9 is currently recognized as a biomarker for the diagnosis of PDAC [34]. In our study, CA19-9 levels demonstrated independent predictive value for hPDAC, consistent with previous findings [37].

In previous literature, lesion size has also been reported as a factor associated with the differentiation between the two conditions, as mass-forming pancreatitis is more likely to present with segmental lesions. This was particularly relevant in our study, where the MFCP group included several cases of autoimmune pancreatitis, which may have influenced the results to some extent [38]. To facilitate the assignment of integer scores for the continuous variable of lesion size, we applied K-cluster analysis and identified a cut-off point of 4.62 cm.

PDAC typically presents as ill-defined tumors with irregular contours due to heterogeneous growth patterns and parenchymal invasion. Well-circumscribed margins are generally characteristic of benign, noninvasive tumors, whereas ill-defined borders usually indicate peritumoral invasion. The irregular shape is attributed to regional variations in tumor proliferation rates and is associated with malignant biological behavior.

Pancreatic duct cut-off emerged as the most heavily weighted predictor in this diagnostic scoring model. As space-occupying lesions, both PDAC and MFCP are likely to cause upstream pancreatic duct dilation, which aligns with our finding that duct dilation was not an independent predictor [23, 39]. Additionally, no statistical difference was observed in the morphology of the dilated pancreatic duct in the univariate analysis. The typical morphology of a dilated pancreatic duct in chronic pancreatitis is beaded expansion, although it can also appear more tortuous and twisted [37, 39, 40]. A similar situation applies to the common bile duct: because both two groups lesion in this study predominantly involved the pancreatic head. Common bile duct dilation and the double duct signs were not significantly different between hPDACs and MFCPs and were therefore not included in the model. Lesions located in the pancreatic head may increase the difficulty of using morphological characteristics for differential diagnosis. In such cases, assessing the degree of lesion fibrosis through pancreatic stiffness measurements may help to effectively and quantitatively indicate the likelihood of benign versus malignant disease [41, 42].

The most heavily weighted factor in the model was pancreatic duct cut-off, and the absence of this feature was defined as either the rat tail sign or visible ductal passage, depending on the pattern of lesion invasion into the pancreatic duct. According to literature reports, the presence of a rat tail sign in pancreatitis or a pancreatic duct-penetrating sign is most often related to the effects of chronic pancreatitis on the formation of fibrotic changes in the pancreatic duct wall and localized compression [37]. By contrast, pancreatic duct involvement in pancreatic cancer typically reflects malignant invasion. Therefore, in clinical diagnosis, evaluation of the differential pattern of pancreatic duct involvement is essential to distinguish between these two diseases.

To enhance practical diagnostic utility, we weighted the predictors in the diagnostic model to create a 17-point scoring system. Comparison of the AUC values between scoring model and clinicoradiological model revealed no significant differences in discriminatory performance, indicating that the scoring system provides a satisfactory and convenient alternative for evaluation.

This study exists several limitations. First, its retrospective design introduces inherent selection bias. Second, dichotomization of quantitative tumor markers may lead to potential information loss. Third, because of the specific enhancement pattern used to define hPDAC and the stringent inclusion criteria for both diseases, the sample size was limited. Fourth, because of the constrained recruitment, external validation is needed in future work to confirm the potential clinical value of this model. Finally, for this atypical enhancement subtype of PDAC, conventional imaging features provided limited diagnostic information, suggesting that future studies may benefit from integrating omics-based approaches for further exploration.

In conclusion, the present study constructed a clinicoradiological diagnostic model for differentiating hPDAC from MFCP incorporating four variables. Weighted scores were assigned to these independent predictive variables to create a 17-point scoring model, which achieved comparable diagnostic performance. Both models demonstrated high accuracy in distinguishing between the two diseases and show potential to offer practical convenience and valuable assistance in clinical diagnosis.

## Electronic supplementary material

Below is the link to the electronic supplementary material.

Supplementary Material 1

## Data Availability

The datasets used and analyzed in the current study are available from the corresponding author on reasonable request.

## Abbreviations

PDAC: Pancreatic ductal adenocarcinoma
hPDAC: High-enhancement pancreatic ductal adenocarcinoma
MFCP: Mass-forming chronic pancreatitis
MRI: Magnetic resonance imaging
CT: Computed tomography
EUS-FNA: Endoscopic ultrasound fine-needle aspiration biopsy
AUC: Area under the ROC curve
ROC: Receiver operating characteristic
CA19-9: Carbohydrate antigen 19 − 9
CEA: Carcinoembryonic antigen
