# Supplementary material for: A scoring model for preoperative differentiation of high-enhancement pancreatic ductal adenocarcinoma from mass-forming chronic pancreatitis
Source: BMC Med Imaging. 2025 Jul 18;25:290. doi: 10.1186/s12880-025-01830-x (PMC12275298; doi:10.1186/s12880-025-01830-x)
Supplement: Supplementary file 1 — Supplementary Material 1 [file 12880_2025_1830_MOESM1_ESM.docx]

**Supplementary materials**

Figure S1. Calibration curve of constructed models.

Figure S2. Imaging manifestations of duct-penetrating sign and pancreatic duct cut-off.

Table S1. Ridge regression results of relevant predictive factors (k=0.30).

Table S2. Multivariate logistic regression of clinical model and imaging model.

**
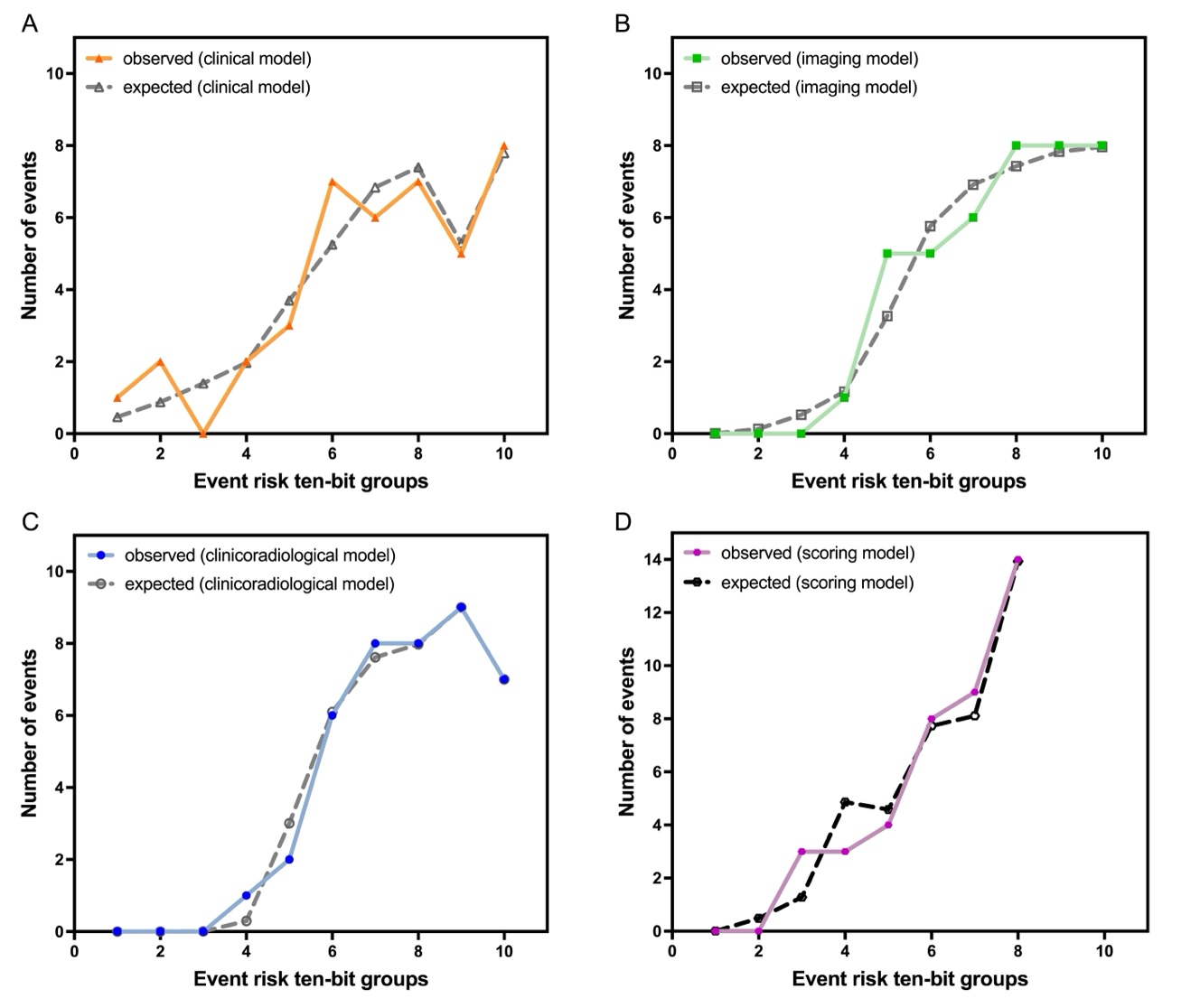
**

**Figure S1. Calibration curve of constructed models. A. The calibration curve of clinical model, (p = 0.561); B. The calibration curve of imaging model (p = 0.833); C. The calibration curve of clinicoradiological model (p = 0.952); D. The calibration curve of scoring model (p = 0.453).**

**
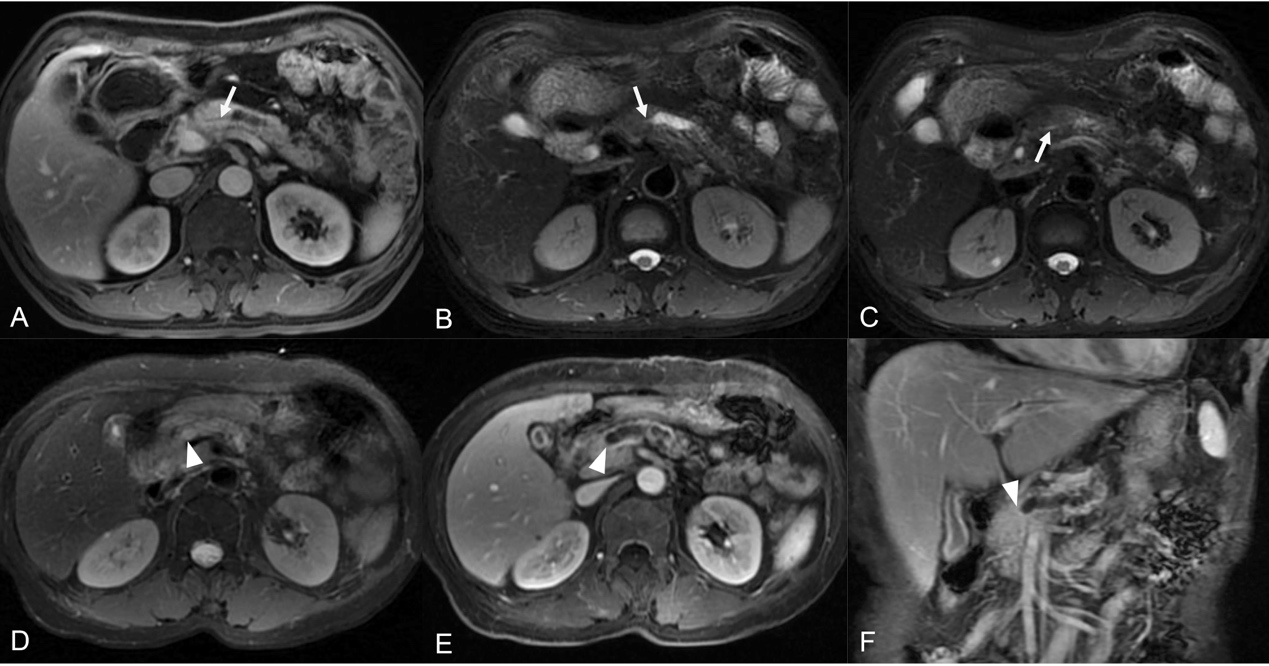
**

**Figure S2. Imaging manifestations of duct-penetrating sign and pancreatic duct cut-off. (A-C) Duct-penetrating sign in a MFCP case, showed a segmental narrowing of the pancreatic duct through the lesion in portal venous phase image and T2-weighted images (white arrow); (D-F) Pancreatic duct cut-off in a hPDAC case, showed a sudden interruption of the pancreatic duct with obstructive dilatation of the upstream duct in T2- weighted image, axial portal venous phase image and coronal portal venous phase image (white arrowhead).**

**Table S1. Ridge regression results of relevant predictive factors (k=0.30).**

|  | Unstandardized Coefficients | | Standardized Coefficients | t | *p* |
| --- | --- | --- | --- | --- | --- |
|  | B | S.E. | Beta |  |  |
| Age | 0.005 | 0.002 | 0.112 | 2.253 | **0.028** |
| Sex | 0.204 | 0.056 | 0.179 | 3.667 | **＜0.001** |
| CEA elevate | -0.010 | 0.060 | -0.009 | -0.175 | 0.862 |
| CA19-9 elevate | 0.283 | 0.049 | 0.283 | 5.748 | **＜0.001** |
| Lesion size | -0.014 | 0.006 | -0.117 | -2.373 | **0.021** |
| Lesion shape | -0.152 | 0.051 | -0.146 | -2.980 | **0.004** |
| Lobulation | 0.252 | 0.052 | 0.240 | 4.831 | **＜0.001** |
| Pancreatic atrophy | 0.113 | 0.051 | 0.111 | 2.218 | **0.030** |
| Lymphadenopathy | 0.102 | 0.050 | 0.102 | 2.056 | **0.044** |
| Dilated pancreatic duct | -0.041 | 0.052 | -0.040 | -0.792 | 0.431 |
| Pancreatic duct cut-off | 0.272 | 0.052 | 0.265 | 5.194 | **＜0.001** |
| Dilated common bile duct | 0.011 | 0.049 | 0.011 | 0.234 | 0.816 |
| Morphology of the dilated common bile duct | 0.039 | 0.026 | 0.072 | 1.485 | 0.142 |
| Constant | -0.218 | 0.146 |  | -1.494 | 0.140 |

**Table S2. Multivariate logistic regression of clinical model and imaging model.**

|  | B | *p* | OR | 95% C.I. for OR |
| --- | --- | --- | --- | --- |
| Clinical model |  |  |  |  |
| Age | 0.081 | **0.008** | 1.085 | 1.022 - 1.151 |
| Sex | 2.117 | **0.006** | 8.308 | 1.857 - 37.171 |
| CEA elevate | 0.633 | 0.405 | 1.882 | 0.425 - 8.333 |
| CA19-9 elevate | 2.75 | **＜0.001** | 15.646 | 4.186 - 58.484 |
| Constant | -6.797 | 0.001 | 0.001 | 0.000 - 0.060 |
| Imaging model | | | | |
| Lesion size | -1.251 | **0.042** | 0.286 | 0.086 - 0.958 |
| Lesion shape | -2.703 | **0.015** | 0.067 | 0.008 - 0.587 |
| Lobulation | 2.648 | **0.018** | 14.125 | 1.561 - 127.797 |
| Pancreatic atrophy | 2.075 | **0.046** | 7.966 | 1.038 - 61.126 |
| Lymphadenopathy | 1.948 | 0.056 | 7.011 | 0.948 - 51.879 |
| Dilated pancreatic duct | -2.318 | 0.063 | 0.099 | 0.009 - 1.131 |
| Pancreatic duct cut-off | 3.514 | **0.004** | 33.571 | 3.079 - 366.047 |
| Dilated common bile duct | -0.254 | 0.858 | 0.776 | 0.049 - 12.416 |
| Morphology of the dilated common bile duct | 0.815 | 0.287 | 2.258 | 0.505 - 10.109 |
| Constant | 0.63 | 0.741 | 1.878 | 0.045 - 78.264 |
